# The Sensitization of Scintillation in Polymeric Composites Based on Fluorescent Nanocomplexes

**DOI:** 10.3390/nano11123387

**Published:** 2021-12-14

**Authors:** Irene Villa, Beatriz Santiago Gonzalez, Matteo Orfano, Francesca Cova, Valeria Secchi, Camilla Colombo, Juraj Páterek, Romana Kučerková, Vladimir Babin, Michele Mauri, Martin Nikl, Angelo Monguzzi

**Affiliations:** 1 Institute of Physics of the Czech Academy of Sciences, FZU, Cukrovarnická 10/112, 162 00 Prague, Czech Republic; paterek@fzu.cz (J.P.); kucerko@fzu.cz (R.K.); babinv@fzu.cz (V.B.); nikl@fzu.cz (M.N.); 2 Eindhoven University of Technology, 5600 MB Eindhoven, The Netherlands; bea.santiagolez@gmail.com; 3 Department of Materials Science, University of Milano-Bicocca, Via R. Cozzi 55, 20125 Milan, Italy; matteo.orfano@unimib.it (M.O.); francesca.cova@unimib.it (F.C.); Valeria.secchi@unimib.it (V.S.); c.colombo103@campus.unimib.it (C.C.); michele.mauri@unimib.it (M.M.); 4 Faculty of Nuclear Sciences and Physical Engineering, Czech Technical University in Prague, Břehová 7, 115 19 Prague, Czech Republic

**Keywords:** scintillation, nanocomposites, energy transfer, metal clusters, hybrid materials, nanoscintillators

## Abstract

The sensitization of scintillation was investigated in crosslinked polymeric composite materials loaded with luminescent gold clusters aggregates acting as sensitizers, and with organic dye rhodamine 6G as the emitting species. The evolution in time of the excited states population in the systems is described by a set of coupled rate equations, in which steady state solution allowed obtainment of an expression of the sensitization efficacy as a function of the characteristic parameters of the employed luminescent systems. The results obtained indicate that the realization of sensitizer/emitter scintillating complexes is the strategy that must be pursued to maximize the sensitization effect in composite materials.

## 1. Introduction

**Figure 1:**
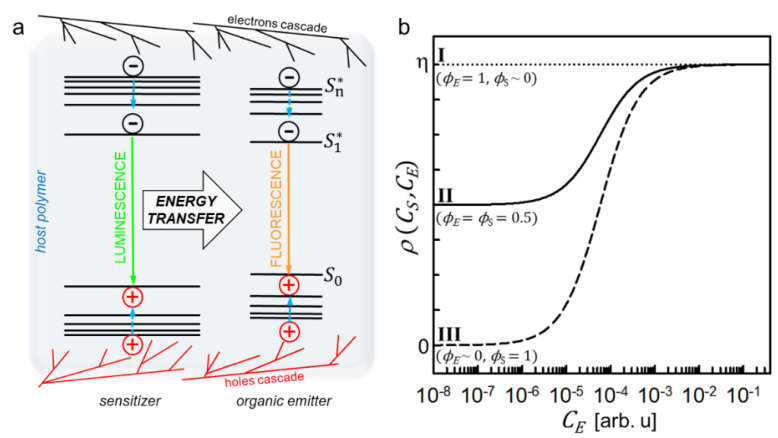
(**a**) Outline of the photophysics involved in the active sensitization of the scintillation process in composite materials based on organic emitters. The dye singlet ground state is indicated by S_0_. Excited dye singlet states are marked with *. Free charges are generated by interaction of the ionizing radiation with the polymer, sensitizer, and emitter moieties. They can recombine directly on emitters or on luminescent sensitizers. The resonance between the X-ray-activated luminescence of sensitizers and emitter absorption enables the sensitization of the emitter luminescence by radiative and non-radiative energy transfer from excited sensitizers. The fluorescent emitters generate the light pulse that will be detected by a photon counter. (**b**) Relative sensitization yield ρ calculated as a function of the energy transfer efficiency ($\phi_{ET}$) between the sensitizer and emitters in the case of dominant charge recombination on emitters (I), competitive recombination on sensitizers and emitters (II) and dominant recombination on sensitizers (III). The parameter η is the relative increment of the energy deposition in the composite by charges ionization with respect to the unsensitized system.

The development of organic materials such as scintillators has become an attractive topic for the scientific community in diverse fields, including biomedical applications, high-energy and nuclear physics, and in homeland security. The sensitization of scintillation in composite plastic materials is therefore pursued to enhance the performance of pure organic scintillators, which present good time response and acceptable scintillation yield, but they typically suffer of re-absorption issues, aggregation problems at high dye concentrations, and intrinsic energy losses due to the presence of optically dark triplet states [1]. In general, the scintillation of organic dyes is activated by direct energy transfer from the polymeric matrix where they are embedded, which is responsible of the primary interaction with the high-energy excitation beam. The inclusion of heavy materials into the composite is exploited to enhance the interaction cross section of the ionizing radiation with the scintillator [2], thus increasing the fraction of deposited energy in the matrix. The sensitization of dye scintillation is then given by the sum of two contributions. The first one is the direct transfer of a fraction of the increased deposited energy from the host matrix to the emitters [3], a process that we can consider as a passive sensitization mechanism. The second contribution relies on an active sensitization process that can be observed if the heavy systems employed to increase the material density are luminescent. Indeed, under specific energetic resonance conditions, the organic emitters can be further activated by a second energy transfer from the heavy components (Figure 1a) [4,5,6]. While the passive sensitization can be seen, in first approximation, as a trivial consequence of the increment of the average scintillator density, the active sensitization requires a deeper analysis to be modelled.

In this work we investigate a model system where the scintillating dye rhodamine 6G (Rh6G) [7,8] is coupled to heavier luminescent gold cluster aggregates, acting as scintillation sensitizers, in a model transparent crosslinked polymer rigid matrix, in order to point out the guidelines for the fabrication of optimized scintillating composites exploiting the sensitized scintillation mechanism. We introduce a system of coupled rate equations that describes the evolution of excited states in time during the scintillation mechanism, thus obtaining an expression of the sensitization efficacy as function of several parameters characteristic of the luminescent materials employed. The reliability of the proposed modelling was verified in a series of composites specimens. The obtained results demonstrate that only a specific composite configuration maximize the sensitization effect.

## 2. Materials and Methods

*Scintillating nanocomplexes preparation*. The organic dye Rhodamine 6G (Rh6G) was purchased from Merck (Darmstadt, Germany, CAS number 989-38-8) and used as is. Au_8_ superstructures were synthesized according to the procedure previously published [9]. Briefly, after thiol-induced etching of gold nanoparticles to obtain individual Au_8_ clusters, the superstructures were formed by networking of the clusters capped with 11-mercaptoundecanoic acid (MUA) ligands. The Au_8_ superstructures/Rh6G complexes were spontaneously formed by mixing the two moieties in solution (see Supplementary Materials, Figures S2–S5).

*Nanocomposites preparation.* The 2-Hydroxyethyl methacrylate (HEMA, Merck (Darmstadt, Germany, CAS no. 868-77-9, MW 130.14 g mol^−1^) was a colorless viscous monomer in liquid phase that readily polymerizes. After the monomer purification from inhibitors, we chose 4,4′-azobis(4-cyanovaleric acid) (Merck (Darmstadt, Germany) CAS number 2638-94-0) as a free radical initiator of the polymerization reaction, that was performed in bulk at room temperature under N_2_ atmosphere in sealed vials. The final composition was obtained by adding 5000 ppm of 4,4′-azobis(4-cyanovaleric acid) (CAS no. 2638-94-0) to a given quantity of HEMA (for 1.5 g of HEMA we added 7.5 mg of activator). All the samples were irradiated with a 365 nm Wood lamp for 1 h. The reaction scheme led to a transesterification process which resulted in the crosslinking of the polymer pHEMA [10]. Given the high solubility of Rh6G in HEMA, a stock solution 10^−2^ M was prepared to be employed in the samples’ series preparation. The Au_8_ superstructures were dispersed in 50 μL of ultrapure water in 4 mL vials, then added to the Rh6G:HEMA solution before polymerization. This step was crucial to obtain a homogeneous dispersion of the Au_8_ superstructures and it allowed the formation of the scintillating Au_8_:Rh6G complexes. Prior to polymerization, the prepared solutions in the sealed vials were purified from oxygen under N_2_ flux for 30 min. The solution was then irradiated with 365 nm UV light for one hour to induce the polymerization. The vials were broken to recover the samples. All composites were prepared with disc geometry with diameter 1 cm and thickness 0.2 cm. The employed crosslinking reaction led to rigid and durable samples with no appreciable residuals of unreacted monomer that can affect the material emission and scintillation properties (Supplementary Materials) [11].

*Photoluminescence (PL) studies.* Absorption spectra were recorded using a Cary Lambda 900 spectrophotometer at normal incidence with Suprasil quartz cuvettes. Steady-state PL spectra were acquired using a Varian Eclipse fluorimeter (bandwidth 1 nm) using quartz cuvettes of 1 cm optical path length. Time-resolved PL spectra were recorded under excitation by a pulsed light-emitting diode at 340 nm (3.65 eV, pulse width 80 ps; EP-LED 340, Edinburgh Instruments). The composites were excited with a pulsed laser at 405 nm (3.06 eV, pulse width 90 ps; EPL-405, Edinburgh Instruments) to avoid direct excitation of the host polymer matrix. Measurements on composites were performed on cylindrical bulk specimens of 1 cm diameter and 0.2 cm thickness.

*Radioluminescence (RL) studies.* Steady-state RL measurements were carried out at room temperature using a homemade apparatus featuring, as a detection system, a liquid nitrogen-cooled, back-illuminated, and UV-enhanced charge-coupled device (Jobin-Yvon Symphony II) combined with a monochromator (Jobin-Yvon Triax 180) equipped with a 100 lines/mm grating. All spectra were corrected for the spectral response of the detection system. RL excitation was obtained by unfiltered X-ray irradiation through a beryllium window, using a Philips 2274 X-ray tube with a tungsten target operated at 20 kV. At this operating voltage, a continuous X-ray spectrum is produced by a Bremsstrahlung mechanism due to the impact of electrons generated through the thermionic effect and accelerated onto a tungsten target. The dose rate was 0.2 Gy/s, evaluated by comparison with a calibrated ^90^Sr-^90^Y beta radioactive source and using optically stimulated luminescence emission from quartz crystalline powder (100–200 μm grains) (Figures S1 and S2).

*Scintillation experiments*. Light yield (LY) was determined by means of amplitude spectroscopy of scintillation pulses with accordance to Ref. [9] and compared to that of a reference BGO crystal measured under the same conditions [12]. Scintillation pulses were excited by ^239^Pu α-radiation (5.2 MeV). The setup for amplitude spectroscopy consisted of a hybrid photomultiplier DEP PPO 475B, spectroscopy amplifier ORTEC 672 (shaping time set to 1 μs) and multichannel analyzer ORTEC 927TM. Ultrafast decays under pulsing X-ray excitation were measured at room temperature using picosecond (ps) X-ray tube N5084 (Hamamatsu Photonics K.K. Shizuoka, Japan) at 40 kV). The X-ray tube was driven by the ps light pulser equipped with a laser diode with a repetition rate up to 1 MHz. The signal was detected by hybrid picosecond photon detector and Fluorohub unit (Horiba Scientific, Kyoto, Japan). The setup instrumental response function FWHM was about 76 ps. The Rh6G scintillation flashes decay curves were detected from the same surface as that excited by X-rays using a 560 nm low pass optical filter (Figure S7). The emission was monitored from the same sample’s surface where it was excited. The deconvolution procedure was applied to the decay curves to calculate true decay times and estimate the pulse rise time (SpectraSolve software package, Ames Photonics, FortWorth, TX, USA).

## 3. Results and Discussion

The setting-up of the equation system employed to model the evolution of the photophysical processes in the systems is based on the fundamental assumption that in a composite scintillator both the heavy sensitizers and the emitter moieties can behave as recombination centers for the high-energy free charges generated by the light–matter interaction [13]. When ionizing radiation or high-energy particles interact with a material, the energy is mainly deposited through ionization [14]. Considering that the created free charges can diffuse up to micrometric volumes before recombination [15], and given that intermolecular distances of the nanosized emitters and sensitizers embedded in the composite are in the order of 10–100 nanometers for concentrations as low as 10^−5^ M, we can assume the rapid diffusion limit for the charge recombination process [16]. This means that the sensitizer and emitter charge capture rates, respectively, in first approximation are proportional to the sensitizer ($C_{sens}$) and emitter ($C_{em}$) concentrations. The evolution in time of the excited species $S^{*}$ for sensitizers and $E^{*}$ for emitters, respectively, can be therefore described by

(1) $$\frac{\partial }{\partial t}\left[S^{*}\right]=\left(\alpha C_{S}-\beta C_{E}-\sigma \right)N-k_{0,S}\left[S^{*}\right]-k_{ET}\left[S^{*}\right]$$

(2) $$\frac{\partial }{\partial t}\left[E^{*}\right]=\left(\beta C_{E}-\alpha C_{S}-\sigma \right)N-k_{0,E}\left[E^{*}\right]+k_{ET}\left[S^{*}\right],$$

where α and β are the free charges capture rate constants for the sensitizer and emitter, respectively, and σ is a loss rate constant that is determined by the matrix characteristics. The rates $k_{0,S}$ and $k_{0,E}$ are the spontaneous recombination rates of the sensitizer and emitter excited states, respectively. N is the total number of electron/hole pairs generated in the host polymer. In general, the N value varies according to the system composition, because both $C_{S}$ and $C_{E}$ can determine the initial density of free charges created through the light–matter interaction.

In steady state conditions, the integrated radioluminescence intensity of the system is given therefore by

(3) $$I_{RL}\left(C_{S},C_{E}\right)=\chi \phi_{E}\left[E^{*}\right]=\chi \phi_{pl}\left(\phi_{S}\phi_{ET}+\phi_{E}\right)N$$

where χ is the instrumental detection efficiency, $\phi_{pl}$ is the quantum yield of the emitter fluorescence and $\phi_{ET}=k_{ET}{\left(k_{ET}+k_{0,S}\right)}^{-1}$ is the yield of the sensitizer-to-emitter energy transfer. The parameters $\phi_{S}$ and $\phi_{E}$ are defined as the sensitizer and emitter charge capture yields, respectively, by

(4) $$\phi_{S}=\frac{\alpha C_{S}}{\alpha C_{S}+\beta C_{E}+\sigma }$$

(5) $$\phi_{E}=\frac{\beta C_{E}}{\alpha C_{S}+\beta C_{E}+\sigma }$$

The sensitization efficiency can be now explicitly expressed as

(6) $$\rho =\left(\phi_{S}\phi_{ET}+\phi_{E}\right)\eta =\varepsilon \left(C_{S},C_{E}\right)\eta ,$$

where $\eta =\frac{N-N^{\prime }}{N^{\prime }}$ is the relative increment of free charges density due to the presence of the heavy component with respect the unsensitized system $N^{\prime }$ loaded with the same emitter concentration. The parameter ε ranges from 0, where the charges are lost by mechanisms competitive to emitter luminescence, to 1, where the total additional energy is properly exploited to activate the emitter luminescence. Therefore, the ρ value indicates the effectiveness of the radioluminescence sensitization as a function of the system composition and the characteristic parameters of the employed materials. The calculated value of ρ as function of the emitter concentration, which sets both $\phi_{E}$ and $\phi_{ET}$, is shown in Figure 1b for the ideal case where σ = 0, i.e., without free charge loss due to the matrix. By considering a composite scintillator with fixed composition, three main scenarios can be identified:

I. The limit case where $\phi_{S}\ll \phi_{E}$, where the sensitizers’ free charges capture ability is negligible with respect to the emitter one independently from $C_{S}$. Despite its luminescence properties, we are in a situation where the sensitizer moiety acts as a passive sensitization component. Any rise of $C_{S}$ will induce a simple growth of η value because of the increment of the overall material density. Notably, this type of sensitization can be effective only with a complete energy transfer from the matrix to the emitters ($\phi_{E\;}$= 1, σ = 0), thus obtaining ρ=η (Figure 1b, dotted line) [17,18]. However, this condition is generally difficult to achieve.

II. If $\phi_{S}≅\phi_{E}$, the deposited energy is equally shared among the two populations. In this case $\rho =\phi_{E}(\phi_{ET}+1)\eta$, thus the effectiveness of the sensitizer-to-emitters energy transfer becomes crucial to recover the energy stored on sensitizers even in the best case with $\phi_{S}$ = $\phi_{E}\;$= 0.5 (Figure 1b, solid line). It is worth noting that this configuration for active sensitization can be advantageous also with a non-complete energy transfer if the η value is at least doubled with respect to the unsensitized case, to balance the charges recombination on sensitizers.

III. In the limit case where $\phi_{S}\gg \phi_{E}$, the energy transfer step becomes more critical, because it is the unique activation channel for the emitter’s luminescence. None of the additional charges recombine on the emitters, thus $\rho =\phi_{S}\phi_{ET}\eta$. Therefore, a poor interaction between emitters and sensitizers can completely make useless the presence of these latter even with $\phi_{S}$= 1, because the activation of the final emission is completely determined by the energy transfer step (Figure 1b, dashed line).

In both case II and case III, the use of highly luminescent sensitizers is strongly recommended to exploit fast non-radiative energy transfer to activate the emitter luminescence while not affecting the scintillator time response. On the contrary, radiative energy transfer can induce a delay of the scintillation emission because of the emission/re-absorption step involved. The composition and structure of the composite scintillator should be therefore optimized to achieve high non radiative transfer rates and 100% transfer yield. Some conditions are then required to reach the maximum sensitization efficacy.

**Figure 2:**
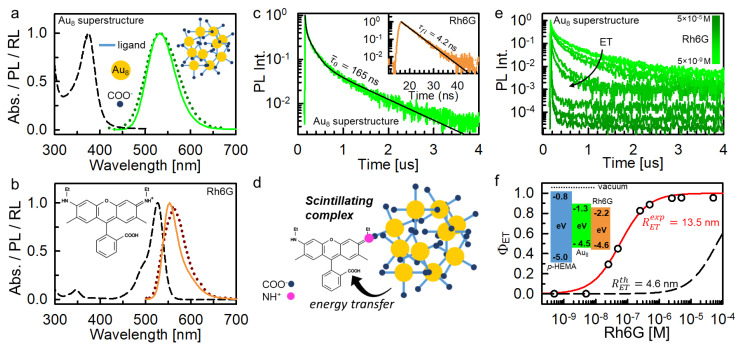
(**a**,**b**) Absorption (dashed line) and photoluminescence (PL, solid lines) of Au_8_ superstructures and rhodamine 6G (Rh6G) in aqueous and ethanol dispersion, respectively. Dotted lines are the corresponding radioluminescence spectra observed under soft X-ray exposure. The insets show the structure of the investigated compounds. (**c**) Time resolved PL at 530 nm spectrum of Au_8_ superstructures dispersion under pulsed excitation at 355 nm. The inset show the Rh6G PL decay at 570 nm. Solid lines are the fit of the PL intensity decays data with multi-exponential and single exponential decay functions, respectively. (**d**) Sketch of the sensitizer/emitter complex formed by Au_8_ superstructures and the Rh6G dye. (**e**) Time resolved PL at 530 nm spectrum of Au_8_ superstructures dispersion under pulsed excitation at 340 nm as a function of the Rh6G concentration in a H_2_O/EtOH mixed solution. (**f**) Au_8_ superstructures-to-Rh6G energy transfer yield $\phi_{ET}$ as a function of the Rh6G concentration. The solid line is the fit of the experimental data with a diffusion-mediated energy transfer process kinetic model with characteristic interaction radius of 13.6 nm. The dashed line is the theoretical efficiency curve calculated from the absorption and PL properties of the investigated compounds with an interaction radius of 4.6 nm. The inset shows the HOMO/LUMO energies expressed in eV for the host polymer p-HEMA, the Au_8_ clusters and the Rh6G dye with respect to the vacuum level.

The role of the sensitizer-to-emitter energy transfer was investigated in a series of composite polymeric scintillators based on a matrix of poly(2-hydroxyethyl methacrylate), poly-HEMA, in which 8-atoms gold clusters aggregated superstructures (Au_8_ superstructures) [19] were employed as sensitizers and the scintillating organic dye Rh6G was employed as final emitters. Figure 2 shows their absorption, luminescence and radioluminescence spectra. The Au_8_ superstructures were selected because of the high atomic number of gold (*Z* = 79) and their large Stokes shift (~0.85 eV), with a first absorption peak in near UV spectral region at 380 nm and a long living emission, with an average lifetime of ${\bar{\tau }}_{0}$ = 165 ns (Figure 2c), peaked at 530 nm (Figure 2a). These features make them good sensitizers candidates because they have a higher interaction cross section with the ionizing radiation compared to organic materials and they do not have any issue related to re-absorption of scintillation light. Their radioluminescence spectrum is identical to the photoluminescence and it is insensitive to exposure to soft X-rays even for large doses (Figure S1).

The employed emitter system is the Rh6G, which possesses a fluorescence peaked at 590 nm with lifetime of 4.2 ns (inset of Figure 2c) and a photoluminescence quantum yield of 0.95 [19]. Crucial for this study, its absorption and emission properties are complementary to those of the Au_8_ superstructures. As showed in Figure 2b, the dye first absorption band peaked at 525 nm is in excellent resonance with the superstructure emission, thus allowing a non-radiative energy transfer mechanism of the Förster type [20] that can be exploited for the sensitization of the emitter luminescence. Moreover, it is worth noting that the highest occupied molecular orbital (HOMO) and the lowest unoccupied molecular orbital (LUMO) energies of both the luminescent systems are similar (Figure 2f, inset) and deeper than the ones of the host polymer [21,22,23,24,25]. This indicates that the electronic affinities of both compounds are comparable and therefore we can reasonably consider valid the assumption α≅β for the charge capture rate constants. We can therefore consider as similar the ability of both sensitizers and emitters to capture scintillation free charges.

By exploiting the interaction between COO^−^ and NH^+^ (Figure 2d), we realized a 1:1 stable sensitizer/emitter complex (Figure S4). We have chosen this scintillating complex configuration because it is particularly favorable according to the cases discussed above. The proximity of the luminescent moieties guarantees indeed a unitary energy transfer yield. Therefore, by employing these sensitizer/emitter complexes the composition of the scintillator can be tuned by keeping the transfer yield constant. Consequently, the system proposed is a good model to investigate the sensitization of the scintillation discussed above. The progressive complexation of the Au_8_ superstructures is monitored by following the evolution of the sensitizer-to-emitter energy transfer dynamic in solution as function of the emitter concentration. Figure 2e shows the time resolved photoluminescence spectrum recorded at 530 nm under pulsed excitation of a dispersion of Au_8_ superstructures (10^−5^ M) and Rh6G solutions. According to the increment of the emitter amount, the lifetime of the residual sensitizer luminescence decreases because of the more efficient energy transfer, whose efficiency $\phi_{ET}$ can be directly estimated from these data (Supplementary Materials, Supplementary Table S1). This analysis gives a highly overestimated Förster interaction radius of $R_{ET}^{exp}$ = 13.5 nm, which is significantly larger than the theoretical expected length $R_{ET}^{th}$ = 4.6 nm (Figure 2e and Supplementary Materials). This reflects the formation of the described complexes, that results in a high transfer efficiency even at extremely low emitter concentrations. Notably, in these complexes the energy transfer is orders of magnitude faster (3.2 GHz, Figure S5) than the spontaneous decay rate of excited superstructures (${\bar{\tau }}_{0}{}^{-1}$ = 6 × 10^−3^ GHz) thanks to the close proximity of the interacting moieties, thus overcoming the diffusion limited kinetics for the Forster mechanism in low viscosity solutions and enhancing the transfer yield. The formation of stable complexes is further demonstrated by time resolved photoluminescence experiments that show an energy transfer rate and yield independent from the complex concentration (Figure S5).

**Figure 3:**
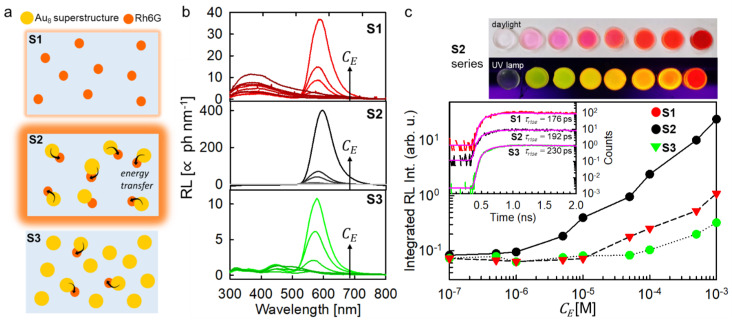
(**a**) Sketch of the composition of the three series (S1, S2, S3) of composites analyzed in this work. Dashed arrows indicate the occurrence of non-radiative energy transfer between in the Au_8_-Rh6G complexes. (**b**) RL spectra of the composite series as function of the emitter Rh6G concentration $C_{E}$ under soft X-rays exposure. (**c**) Digital picture of the most performing S2 series under daylight (top) and UV excitation and integrated RL intensity for the series of composites investigated (bottom). The inset of the bottom panel shows the rise of scintillation pulse of the sample with the highest loading level for each series recorded at 630 nm. The uncertainty of the rise times values is assessed at 50 ps.

Three series of scintillating composites were fabricated with different loading level of luminescent compounds, as sketched in Figure 3a (Supplementary Materials). The first series (S1) contains only the emitter Rh6G, and it works as a reference unsensitized scintillator. The second series (S2) embeds the sensitizers/emitter complexes. The third one (S3) was prepared by including a large and fixed number of individual sensitizers (10^−2^ M) while varying the number of scintillating complexes. In all compositions, the Rh6G keeps its emission properties (Figure S6). Figure 3b depicts the RL spectra of the composite series recorded under the same experimental conditions. The S1 series shows the UV luminescence from poly-HEMA peaked at 360 nm, which intensity decreases with the increment of the emitter concentration. This indicates a better harvesting of the energy of the interacting ionizing radiation by the included R6Gh molecules. When a concentration of $C_{E}$ = 10^−5^ M is reached, the UV emission is switched off, and the Rh6G emission appears with an intensity that gradually increases with the emitter concentration until a $C_{E}$ = 10^−3^ M is reached. A further increment of the dye concentration is not possible due to evident phase segregation of the dyes with respect to the host.

The S2 scintillator series shows a significantly better performance. No UV emission can be detected event at very low loading amounts. This suggests an enhanced ability of Au_8_ superstructures to harvest the deposited energy with respect to the dye, most probably due to the resonance between the polymer luminescence and superstructures absorption that favors direct energy transfer from the matrix. The radioluminescence intensity reaches the maximum value observed in the S1 series with a $C_{E}$ = 5 × 10^−5^ M, which is more than one order of magnitude lower than the one employed for the unsensitized case, suggesting an effective sensitization of the Rh6G luminescence. With the highest loading level of $C_{E}$ = 10^−3^ M, the composite shows a LY of 80 ph MeV^−1^ when excited by ^239^Pu α-radiation (Supplementary Table S2) and an enhanced radioluminescence integrated intensity that indicates a sensitization efficiency of ρ ~ 25 (Figure 3c). It is worth noting that in addition to the improvement of the scintillation performance, the stable structural coupling of the active moieties has several important consequences. First, it makes the energy transfer rate and yield independent from $C_{E}$, that is useful for the technological perspective. Indeed, considering the typical Förster radii values of 2–3 nm [20], a huge amount $C_{E}\geq$ 10^−2^ M of the acceptor/emitter moieties should be included to maximize the transfer in disordered solid systems such has polymeric hosts, where molecular excitons cannot diffuse freely and the transfer is limited to the closest acceptor units [26]. In other words, any configuration with separated sensitizers and emitters would result in a lower sensitization efficiency. Second, this architecture avoids emitters aggregation by enabling a better management of the scintillator composition at high loading levels pivotal for fabrication of efficient detectors with good optical quality by handling a single moiety [27]. Third the coupling sensitizer and emitter at a fixed close distance (≤1 nm) enables a fast energy transfer. Specifically, the transfer rate of 3.2 GHz is significantly larger than the spontaneous recombination rate of the emitter $k_{fl}$ = ${\left(\tau_{fl}\right)}^{-1}$ = 0.24 GHz. This result is pivotal to preserve the time response of the scintillator, as demonstrated by the scintillation data reported in the inset of Figure 3c. Both the S1 and S2 composites show indeed almost identical scintillation activation dynamics, with a rise time of the pulse at around 190 ps unaffected by the fast energy transfer process involved in S2.

The crucial role of the energy transfer optimization is further highlighted by the results obtained on the S3 composite series. Here, the scintillating complexes and the isolated Au_8_ superstructures were added to the composites in order to increase the amount of the Rh6G emitters by keeping the total $C_{S}$ value as large as 10^−2^ M, i.e., significantly higher than the maximum value employed in the best sample of the S2 series. In such a way, the η value should be significantly higher than for the S2 scintillators. However, the radioluminescence intensity is lower also with respect to unsensitized S1 series for any composition thus indicating that most of the deposited energy is lost by competitive channels as suggested by the presence of a residual emission from isolated Au_8_ superstructures in the blue/green spectral region (Figure 3b, bottom panel). This result can be explained considering that the large number of sensitizers amplifies η, but also makes their charges capture yield dominant with respect to the emitter one ($\phi_{S}\gg \phi_{E}$, case III). Therefore, considering that even in the best composition only 1 tenth of sensitizers are coupled to emitters, the poor energy transfer efficiency results in a significant lower scintillation performance despite the higher amount of deposited energy.

## 4. Conclusions

**Figure 4:**
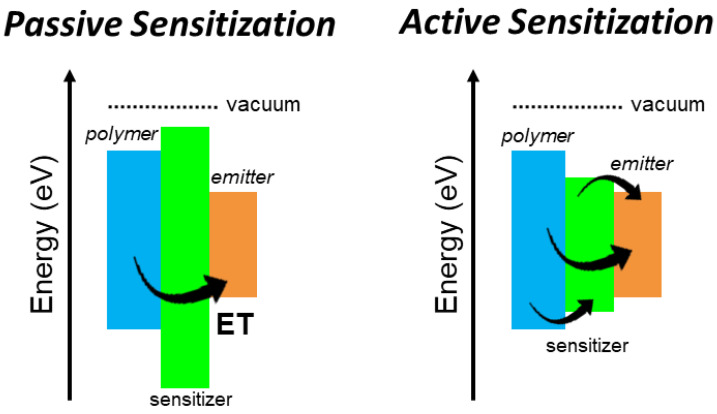
Outline of the energetics in an example multicomponent composite scintillator exploiting passive and active sensitization, respectively. The black arrow highlights the energy transfer processes (ET) that activate the luminescence of the emitter moiety.

In conclusion, the obtained findings demonstrate that active sensitization in multicomponent composite materials is possible by exploiting non-radiative energy transfer from high-density scintillators to efficient and fast molecular scintillators and it can be used to overcome the limits of passive sensitization strategy, such as the presence of inactive sensitizers that can detrimentally compete with the energy sharing mechanism in the system, thus reducing the final light output from the emitters (Figure 4).

In our study, we have emphasized that the choice of the sensitizers and emitters with matching electronic properties to be embedded in the polymeric host, is crucial for the optimization of the sensitizers–emitters energy transfer and the accomplishment of higher sensitization efficiency with respect to the case of the common plastic single component scintillators. Besides, we have highlighted that the use of sensitizer/emitter complexes where the two components are linked together in a unique architecture is particularly interesting from the manufacturing perspective, since the loading of the polymer host will be easier with respect to the case where two separated active species should be handled. The mechanism of active sensitization in scintillating sensitizer/emitter complexes enables to overcome the difficulties to manage the energy transfer process in the solid-state and to partially limit emitters aggregation issues. This architecture is quite versatile and allows to control the system timing performances. By adapting the energetic resonance and the oscillator strength of the electronic transition involved, by decorating the sensitizers with more than one emitter, or by modifying the sensitizer to emitter distance with modulable ligand systems [28,29], the tuning of the ET rate and therefore the control of the rise time of the scintillation signal are achievable and targeted for specific application requests. To complete the description of scintillator in composite polymer materials, future works should be focused on the investigation of any potential correlation between the properties of the crosslinked polymer matrix, such as the crosslink density [30], and the scintillation performances. Finally, it is worth noting that composite scintillators, similar to the ones discussed in this work, would benefit from dedicated studies investigating the punctual interaction of the ionizing radiation with nanosized dense objects to point out potentially beneficial effects on the scintillation yield with respect to a classical homogeneous material.

## Data Availability

The data that support the plots within this paper and other findings of this study are available from the corresponding authors upon reasonable request.

## Supplementary Materials

The following are available online at https://www.mdpi.com/article/10.3390/nano11123387/s1. Figure S1: Radioluminescence (RL) spectrum of a Au_8_ superstructures, Figure S2: Spectral overlap between the Rh6G absorption (dashed line) and Au_8_ superstructure photoluminescence, Figure S3: Time resolved PL spectrum at 530 nm of Au_8_ superstructures (10^−3^ M) and Rh6G (10^−7^ M) solutions, Figure S4: Z-potential measurements, Figure S5: Time resolved photoluminescence spectra of Au8 superstructures (10^−2^ M) and Rh6G (10^−7^ M) composite in p-HEMA. Figure S6: Time resolved PL spectrum at 610 nm of S1, S2 and S3 composites under pulsed laser excitation at 532 nm. Figure S7: Scintillation pulse of the sample with the highest loading level for the nanocomposite series S2. Figure S8: pHEMA structural analysis. Table S1: Fit parameters employed to analyze the time resolved luminescence, Table S2: pHEMA swelling test data. Table S3: LY data report.
