# Supplementary material for: The Sensitization of Scintillation in Polymeric Composites Based on Fluorescent Nanocomplexes
Source: Nanomaterials (Basel). 2021 Dec 14;11(12):3387. doi: 10.3390/nano11123387 (PMC8704871; doi:10.3390/nano11123387)
Supplement: Supplementary file 1 [file nanomaterials-11-03387-s001.zip › nanomaterials-1480261-supplementary.pdf]

Supporting Information for

The Sensitization of Scintillation in Polymeric Composites Based on Fluorescent Nanocomplexes

Irene Villa [1,*,†], Beatriz Santiago Gonzalez [2,†], Matteo Orfano [3], Francesca Cova [3], Valeria Secchi [3], Camilla Colombo [3], Juraj Páterek [1,4], Romana Kučerková [1], Vladimir Babin [1], Michele Mauri [3], Martin Nikl [1] and Angelo Monguzzi [3,*]

[1] Institute of Physics of the Czech Academy of Sciences, FZU, Cukrovarnická 10/112, 162 00 Prague, Czech Republic; paterek@fzu.cz (J.P.); kucerko@fzu.cz (R.K.); babinv@fzu.cz (V.B.); nikl@fzu.cz (M.N.)
[2] Eindhoven University of Technology, 5600 MB Eindhoven, The Netherlands; bea.santiagolez@gmail.com
[3] Dipartimento di Scienza dei Materiali, Università degli Studi Milano-Bicocca, via R. Cozzi 55, 20125 Milano, Italy; matteo.orfano@unimib.it (M.O.); francesca.cova@unimib.it (F.C.); Valeria.secchi@unimib.it (V.S.); c.colombo103@campus.unimib.it (C.C.); michele.mauri@unimib.it (M.M.)
[4] Faculty of Nuclear Sciences and Physical Engineering, Czech Technical University in Prague, Břehová 7, 115 19 Prague 1, Czech Republic
* Correspondence: villa@fzu.cz (I.V.); angelo.monguzzi@unimib.it (A.M.)
† These authors contributed equally.

Methods

Nanoscintillators investigation.

Time resolved photoluminescence experiments (Figs.S3, S5) and Z-potential analysis (Fig.S4) demonstrate the formation on stable complexes. The analysis of the gold-to-dye Forester energy transfer dynamics as a function of the Rh6G indicates that a complete transfer is achieved when the relative ratio of individual Au8 clusters to Rh6G molecules in solution is 10:1. Considering that a Forster type interaction is involved, we can reasonably assume that a complete transfer can be observed when each energy donor system (the Au_9 superstructure) is attached to an acceptor system (the Rh6G molecule). This suggest that each Au_8 superstructure contains on average 10 gold Au_8 clusters. Z-potential measurements have been performed with Malvern Zetasizer nano using Malvern DTS 1070 dedicated cuvettes.

Time resolved data analysis.

The time resolved photoluminescence (PL) decay spectra has been reproduced with an analytical multi-exponential function

$$I_{PL}(t) \propto \sum_i A_i e^{-\left(t/\tau_i\right)} . \qquad \text{(S1)}$$

The parameters used for the fitting procedure are reported in Table 1. The emission lifetime τ_{avg} has been calculated as the weighted average of the characteristic decay time for each i-exponential function using by

$$\tau_{avg} = \frac{\sum_i A_i \tau_i}{\sum_i A_i} . \qquad \text{(S2)}$$

The energy transfer yield ϕ_{ET} vs. C_E has been calculated from the time resolved data showed in Fig. 2d by using the relationship

$$\phi_{ET} = 1 - \frac{\tau_{avg}}{\bar{\tau}_0} . \qquad \text{(S3)}$$

where is the spontaneous lifetime of the Au_8 superstructures (Fig.2c in the main text).

Table S1. | Fit parameters employed to analyze the time resolved luminescence recorded at 500 nm on Au8 superstructres:Rh6G solutions as function of the Rh6G concentration (C_E).

C_E [M]	A1	τ_1 (ns)	A2	τ_2 (ns)	A3	τ_3 (ns)	τ_{avg}(ns)	ϕ_{ET}
0	0.50	16.1	0.36	160.2	0.10	1000.0	165.1 ($\bar{\tau}_0$)	0.00
5.0e-10	0.49	16.7	0.37	158.2	0.10	1002.0	166.1	0.00
5.0e-9	0.55	20.0	0.36	158.0	0.09	917.4	152.0	0.08
2.5e-8	0.57	14.3	0.34	117.4	0.08	662.2	101.0	0.39
5.0e-8	0.60	12.5	0.31	100.0	0.08	529.1	80.8	0.51
2.5e-7	0.70	3.4	0.24	33.3	0.06	175.1	20.9	0.87
5.0e-7	0.88	4.5	0.11	50.0	3.15e-3	2358.5	16.9	0.90
2.5e-6	1	5.3	--	--	--	--	5.3	0.97
5.0e-6	1	5.3	--	--	--	--	5.3	0.97
5.0e-5	1	5.6	--	--	--	--	5.6	0.97

The energy transfer rate k_{ET} can be therefore derived from experimental data using

$$\phi_{ET} = \frac{k_{ET}}{k_{ET}+(\bar{\tau}_0)^{-1}}\,. \qquad (S4)$$

In the rapid diffusion limit, the rate k_{ET} is calculated as

$$k_{ET} = \frac{4\pi C_E R_{fs}^6}{3\bar{\tau}_0 a^3}, \qquad (S5)$$

where R_{fs} is the Förster radius that characterizes this donor/acceptor pair and a = 1.1 nm is the minimum intermolecular center-to center distance. [1] by coupling Eq. S4 and S5 to fit of the experiment data on ϕ_{ET} we obtain a R_{fs}= 13.5 nm, which has been compared to the theoretical radius R_{fs}^* = 4.6 nm calculated form the absorption and PL properties of the emitters and sensitizer moieties by

$$R_{fs}^* = 0.211\left[J(\lambda)\theta^2 n^{-4}\mathrm{QY}_{\mathrm{Au_8}}\right]^{1/6}, \qquad (S6)$$

where θ^2 = 2/3 is the random orientation factor between the transition momentum of the involved electronic transitions on the donor and acceptor systems, n = 1.33 is the refraction index and $\mathrm{QY}_{\mathrm{Au_8}}$= 0.045 is the Au_8 superstructures photoluminescence quantum yield. The overlap integral $J(\lambda)$ is calculated form experimental data as

$$J(\lambda) = \frac{\int PL(\lambda)\varepsilon(\lambda)\lambda^4 d\lambda}{\int PL(\lambda)d\lambda}\,, \qquad (S7)$$

where is the photoluminescence spectrum of the sensitizer and $\varepsilon(\lambda)$ is the molar extinction coefficient of the emitter Rh6G.[2]

Composite analysis. While most radical polymerizations with acrylates produce linear polymers, it is known that HEMA can also self-crosslink due to transesterification of the growing chains.[3] We first verify the cross linking of the pHEMA by checking its solubility in ethanol (EtOH), which is a good solvent for the monomer. The pHEMA doesn't dissolve in EtOH, its hygroscopic character enables the polymer to adsorb the solvent giving rise to the so-called swelling phenomenon, as shown by the data reported in Table S2. No mass is removed by EtOH, thus suggesting the complete cross linking.

Table S2. | Weight of pHEMA sample before and after wetting in EtOH.

	Mass (mg)
Dry sample	28.7
After EtOH treatment	31.8

After 20 hours from EtOH treatment	29.2
After 2 days	28.7

Second, the pHEMA doesn't dissolve even in chloroform, a very aggressive solvent, and the dry weight of the sample after the treatment does not change. Lastly, to further verify the topological organization of the polymer, we applied a selection of time domain NMR (TD-NMR) sequences, often used to undestand the morphology and dynamics in rubber and plastics materials. [4] The most informative result is obtained by measuring the rigid fraction (RF) as function of the temperature using the MSE refocusing block. As seen in Fig. S8, which presents a selection of the experiments, the FIDs acquired over a wide range of temperatures are remarkably similar, and indicate a sample that at the microscopic level is highly constrained, in agreement with the hypothesis of high crosslinking. Experiments were performed on a Bruker Minispec mq20 with the sequence and the parameters described in [4]. Each single FID was fitted with a two component model that distinguishes a rigid fraction with gaussian relaxation, and a mobile fraction associated to exponential relaxation. The result of the analysis is shown in Fig.S8b; at low temperature, the material is fully immobilized (RF=85%), except for residual mobility possibly due to chain ends and side chains. Upon reaching the value reported in literature for the glass transition of bulk PHEMA (373K) the polymer chains increase their local mobility, as demonstrated by the slow decline of rigid fraction. Usually, such slow decline spanning 50 or more K, is associated to the glass transition of homogeneous samples, while samples characterized by complex multiphase morphologies produce multistep evolution of the RF. [5]During the fitting we also noticed that even the apparent T2 associated to the mobile region does not surpass 0.1 ms: compared to typical values for rubbers, that are in the range of ms to tens of ms, this is a further indication that even the mobile part far above the glass transition is strongly constrained, probably by the presence of a dense network of crosslinks.

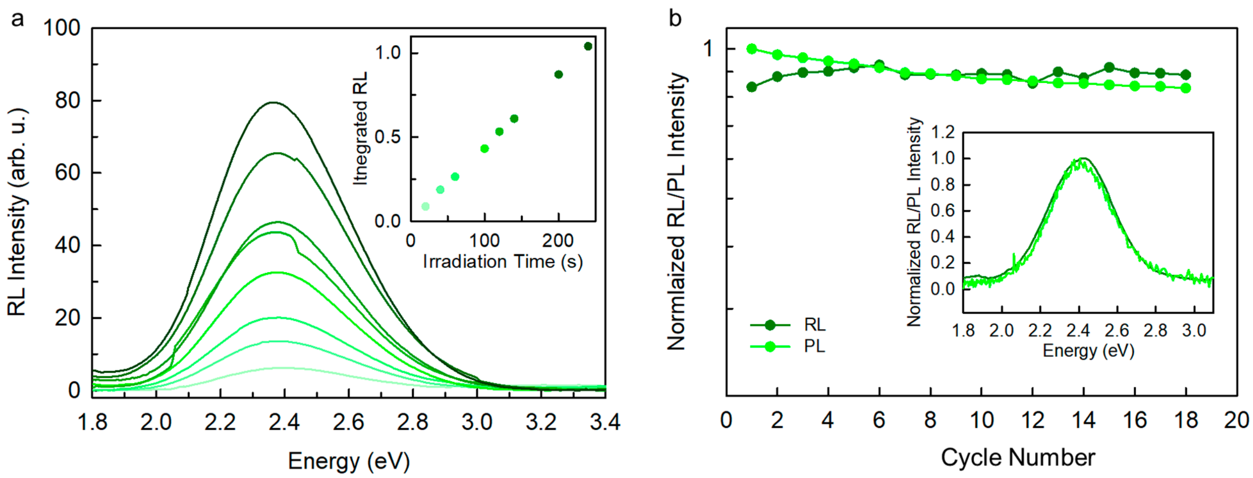

Figure S1. (a) Radioluminescence (RL) spectrum of a Au_8 superstructures drop casted film as a function of the irradiation time under 0.2 Gy s^{-1} at room temperature. The inset is the integrated RL value that shows a liner dependency on the irradiation time. (b) RL and photoluminescence (PL) integrated intensity under X-ray exposure as a function of the irradiation cycle number (1 cycle = 30 s). The inset shows an RL and a PL reference spectrum.

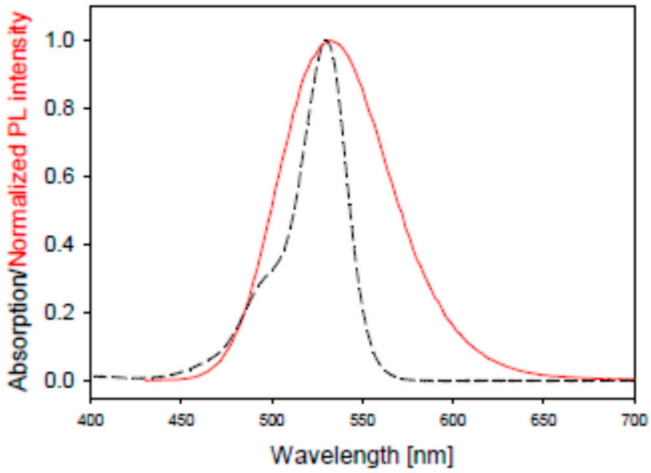

Figure S2. Spectral overlap between the Rh6G absorption (dashed line) and Au_8 superstructure photoluminescence (PL, solid lines).

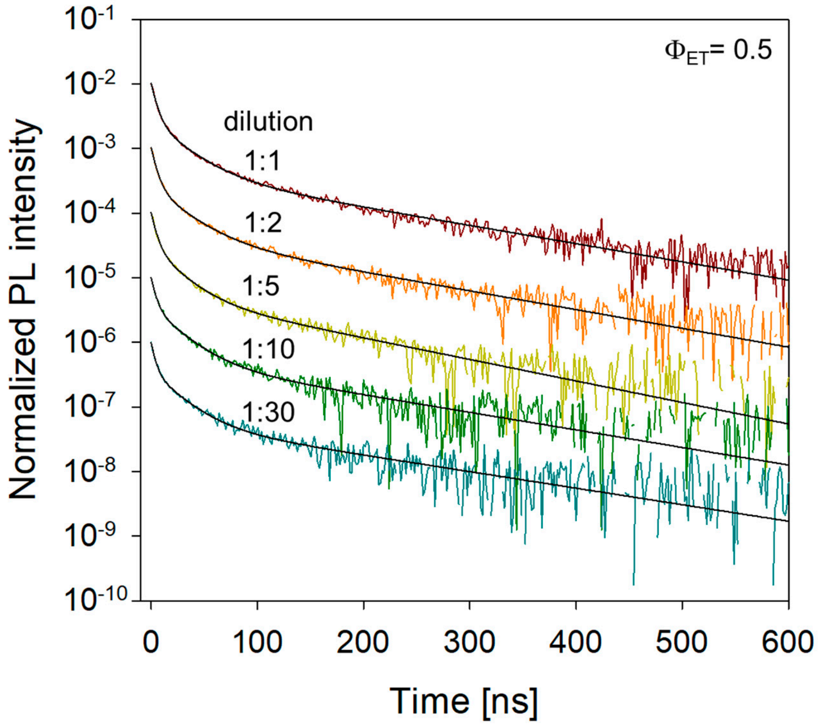

Figure S3. Time resolved PL spectrum at 530 nm of Au_8 superstructures (10^{-3} M) and Rh6G (10^{-7} M) solutions. The energy transfer yield is ϕ_{ET}= 0.5. calculated from the analysis of the PL decay. Non changes in the ϕ_{ET} value are observed upon dilution of the sample down to 1:30, indicating the presence of a stable sensitizer/emitter complex where the energy transfer occurs and it is insensitive to the dilution level. The long-times tail of the PL signal is ascribed to residual un-complexed Au_8 superstructures that survive because of the low concentration of Rh6G dye employed.

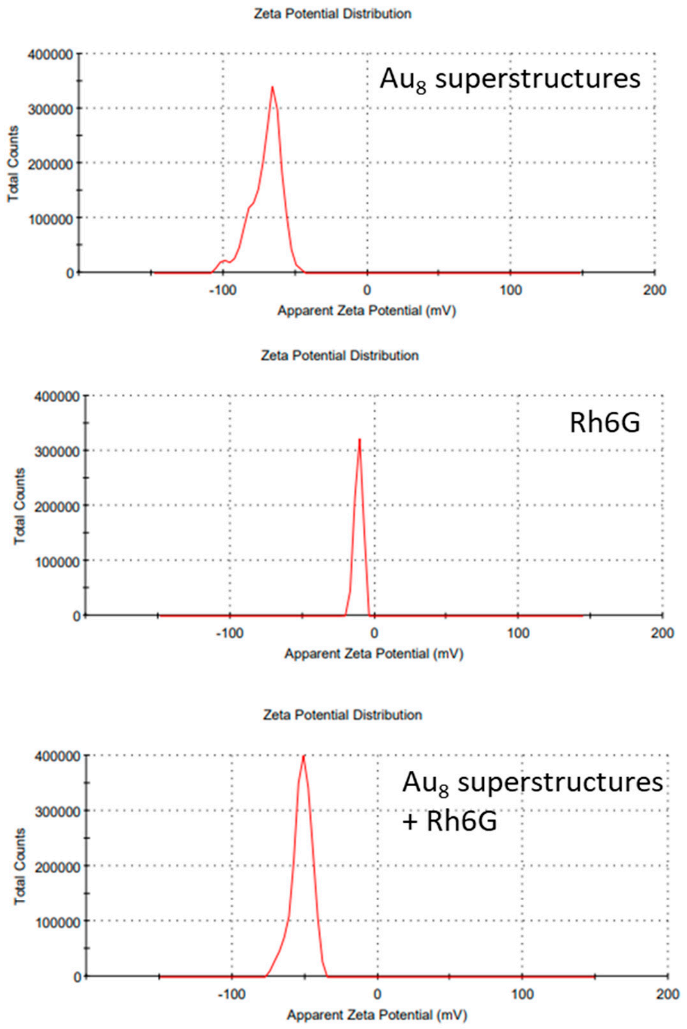

	Surf. Z potential (mV)	Z deviation (mV)	St. Dev. (mV)
Au_8 superstructures	-67.3	11.0	9.50
Rh6G	-11.4	4.24	2.77
Au_8 superstructures + Rh6G	-52.1	8.25	6.90

Figure S4. Z-potential measurements of Au_8 superstructures (5×10^{-4} M), Rh6G (5×10^{-5} M) and Au_8 superstructures:Rh6G mixed solution in water). The reduction of the surface potential of gold superstructures indicates the partial passivation of the carboxylic groups by the Rh6G molecule, thus suggesting the formation of a stable complex as demonstrated by the dilution independent PL properties showed in Fig. S5.

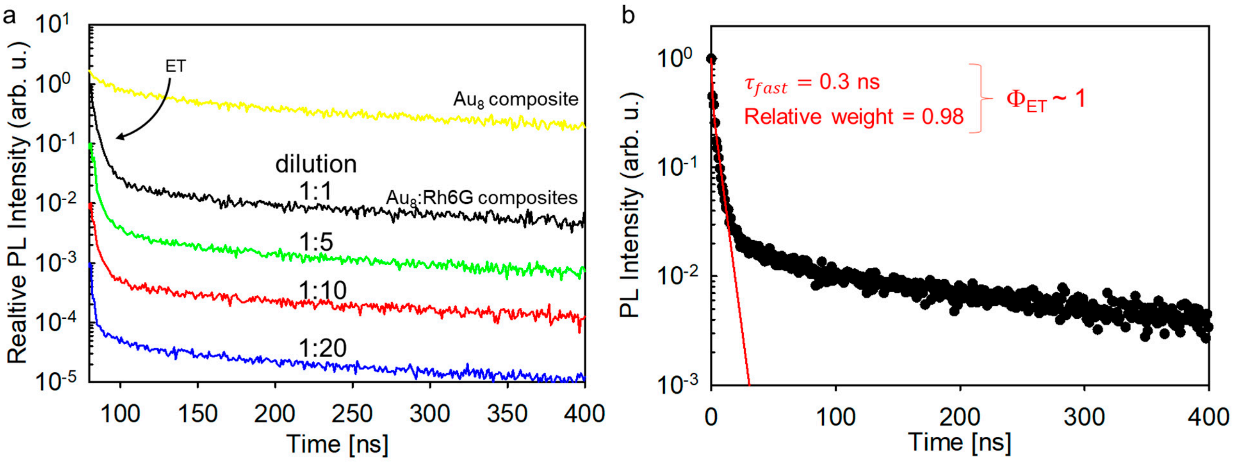

Figure S5. (a) Time resolved PL spectrum at 530 nm of Au_8 superstructures (10^{-2} M) and Rh6G (10^{-7} M) composite in p-HEMA. The energy transfer yield is ϕ_{ET}= 1. calculated from the analysis of the PL decay in panel (b). Time resolved PL spectrum at 530 nm of Au_8 superstructures (10^{-2} M) and Rh6G (10^{-3} M). Here the solid line is the fit of the fast decay component with a single exponential decay function with characteristic time τ_{fast}. Non changes in the energy transfer kinetics are observed upon dilution of the sample down to 1:20, indicating the presence of a stable sensitizer/emitter complex where the energy transfer occurs and it is insensitive to the dilution level also in the solid state. The long-times tail of the PL signal is ascribed to a negligible residual fraction of un-complexed Au_8 superstructures (2%).

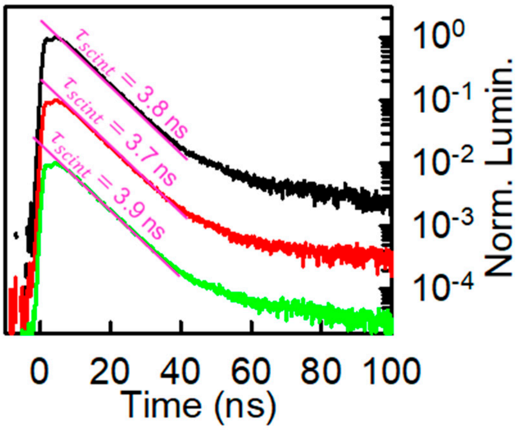

Figure S6. (a) Time resolved PL spectrum at 610 nm of S1 (red), S2 (black) and S3 (green) composites under pulsed laser excitation at 532 nm. Solid lines are the fit of data with a single exponential decay function. No evidence of emission quenching is detected. We can assume that the Rh6G dye preserve its typical high emission yield.

Table S3. Light yield under ^{239}Pu α-radiation and 1 μs shaping time. BGO crystal used as a reference.

Sample	L.Y. (ph/MeV)
1G-gold cluster+rhoda-mine 6G	80
BGO reference	1 010

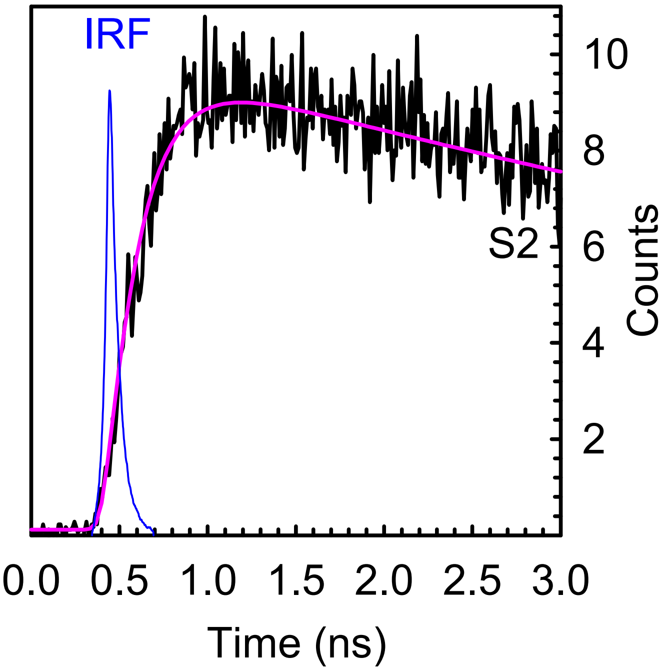

Figure S7. Scintillation pulse of the highest loading level sample from **S2** series. The blue plot is instrumental response function (IRF) employed in the convolution analyses algorithm to evaluate the pulse rise time. Considering the FWHM of the IRF, we can estimate a conservative uncertainty on the rise time value of about 50 ps.

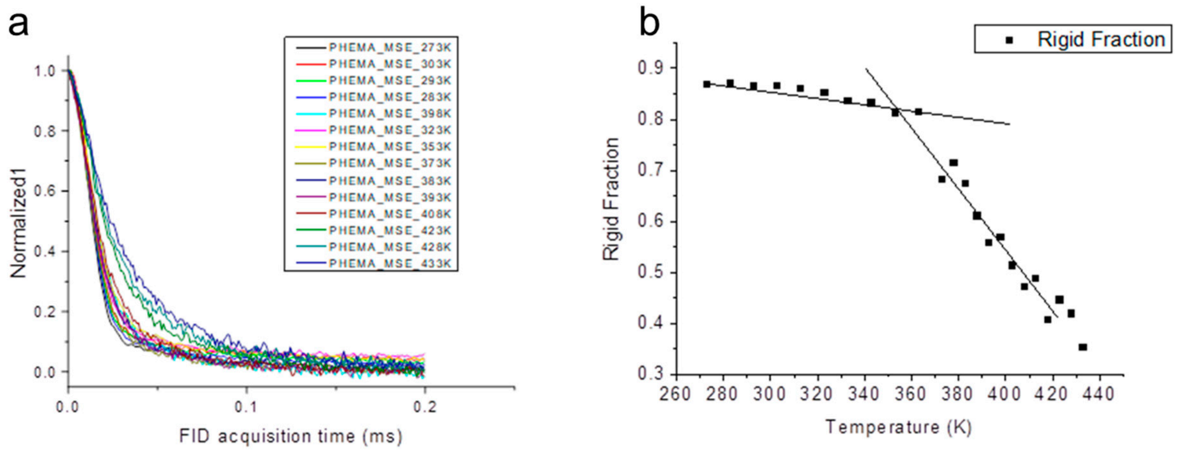

Figure S8. a. Time-domain NMR free-induction decay (FID) of the pHEMA as a function of the temperature. b. Rigid fraction in the materials estimated form the FID data analysis.

Supplementary References

1. Thomas, D. D.; Carlsen, W. F.; Stryer, L., Fluorescence energy transfer in the rapid-diffusion limit. *Proceedings of the National Academy of Sciences* **1978,** *75* (12), 5746.
2. Lakowicz, J. R., *Principles of fluorescence spectroscopy*. Springer science & business media: 2013.
3. Robinson, K.; Khan, M.; de Paz Banez, M.; Wang, X.; Armes, S., Controlled polymerization of 2-hydroxyethyl methacrylate by ATRP at ambient temperature. *Macromolecules* **2001,** *34* (10), 3155-3158.
4. Besghini, D.; Mauri, M.; Simonutti, R., Time Domain NMR in Polymer Science: From the Laboratory to the Industry. *Applied Sciences* **2019,** *9* (9), 1801.
5. Bonetti, S.; Farina, M.; Mauri, M.; Koynov, K.; Butt, H.-J.; Kappl, M.; Simonutti, R., Core@shell Poly(n-butylacrylate)@polystyrene Nanoparticles: Baroplastic Force-Responsiveness in Presence of Strong Phase Separation. *Macromolecular Rapid Communications* **2016,** *37* (7), 584-589.
